# Multiple sclerosis incidence rate in southern Iran: a Bayesian epidemiological study

**DOI:** 10.1186/s12883-021-02342-1

**Published:** 2021-08-10

**Authors:** Naeimehossadat Asmarian, Zahra Sharafi, Amin Mousavi, Reis Jacques, Ibon Tamayo, Marie-Abèle Bind, Marzie Abutorabi-zarchi, Mohammad Javad Moradian, Sadegh Izadi

**Affiliations:** 1 Anesthesiology and Critical Care Research Center, Shiraz University of Medical Sciences, Shiraz, Iran; 2 Health Promotion Research Center, Zahedan University of Medical Sciences, Zahedan, Iran; 3 Department of Epidemiology & Biostatistics, School of Health, Zahedan University of Medical Sciences, Zahedan, Iran; 4 Department of Educational Psychology and Special Education, College of Education, University of Saskatchewan, Saskatoon, Canada; 5 Service de Neurologie, Centre Hospitalier Universitaire, Hôpital de Hautepierre, 1, avenue Molière, 67200 Strasbourg, France; 6 Department of Statistics, Faculty of Arts and Science, Harvard University, Cambridge, MA USA; 7 Department of neurology, Faculty of Medicine, Shahid Sadoughi University of Medical Sciences, Yazd, IR Iran; 8 Trauma Research Center, Shahid Rajaee (Emtiaz) Trauma Hospital, Shiraz University of Medical Sciences, Shiraz, Iran; 9 Clinical Neurology Research Center, Shiraz University of Medical Sciences, Shiraz, Iran

**Keywords:** Bayesian spatio-temporal model, Multiple sclerosis, Incidence rate

## Abstract

**Background:**

Multiple Sclerosis (MS) remains to be a public health challenge, due to its unknown biological mechanisms and clinical impacts on young people. The prevalence of this disease in Iran is reported to be 5.30 to 74.28 per 100,000-person. Because of high prevalence of this disease in Fars province, the purpose of this study was to assess the spatial pattern of MS incidence rate by modeling both the associations s of spatial dependence between neighboring regions and risk factors in a Bayesian Poisson model, which can lead to the improvement of health resource allocation decisions.

**Method:**

Data from 5468 patients diagnosed with MS were collected, according to the McDonald’s criteria. New cases of MS were reported by the MS Society of Fars province from 1991 until 2016. The association between the percentage of people with low vitamin D intake, smoking, abnormal BMI and alcohol consumption in addition to spatial structure in a Bayesian spatio-temporal hierarchical model were used to determine the relative risk and trend of MS incidence rate in 29 counties of Fars province.

**Results:**

County-level crude incidence rates ranged from 0.22 to 11.31 cases per 100,000-person population. The highest relative risk was estimated at 1.80 in the county of Shiraz, the capital of Fars province, while the lowest relative risk was estimated at 0.11 in Zarindasht county in southern of Fars. The percentages of vitamin D supplementation intake and smoking were significantly associated with the incidence rate of MS. The results showed that 1% increase in vitamin D supplementation intake is associated with 2% decrease in the risk of MS and 1% increase in smoking is associated with 16% increase in the risk of MS.

**Conclusion:**

Bayesian spatio-temporal analysis of MS incidence rate revealed that the trend in the south and south east of Fars province is less steep than the mean trend of this disease. The lower incidence rate was associated with a higher percentage of vitamin D supplementation intake and a lower percentage of smoking. Previous studies have also shown that smoking and low vitamin D, among all covariates or risk factors, might be associated with high incidence of MS.

## Background

Multiple Sclerosis (MS) is an autoimmune disorder in which the central nervous system myelin is attacked. It results in focal lesions and clinical symptoms [1, 2]. The disease is classified into four different courses: a) Relapsing- Remitting (RR) MS, the most common type, b) Secondary Progressive (SP); c) Primary Progressive (PP) MS, accounts for only 10% of the cases; and d) Progressive Relapsing (PR) MS [3]. Worldwide, more than 2.3 million individuals are affected by these different types of MS per year [4–6]. The distribution of MS increases with increasing distance from the Equator with some exceptions. Canada, Norway, and Sweden have some of the highest prevalence rates of MS in the world, reported in 2013. However, there are exceptions; some countries farther away from the equator, such as Russia, have low prevalence. On the other hand, other countries which are closer to the equator, for instance Australia, show a high prevalence of the disease [5, 6]. Epidemiological studies based on the geographical regions shows that the prevalence of the disease ranged from 5.30 to 74.28 per 100,000 individuals in Iran, which is not evenly distributed across different regions [7]. Fars province, with 72.10/100,000 (116.50 in females and 28.30 in males) prevalence rate in 2013, is one of the provinces with high risk of this disease. The mean annual incidence rate was 5.2/100,000 from 2002 until 2012 in this province [8].

Environmental factors and lifestyle approaches including vitamin D deficiency, obesity, alcohol consumption, and cigarette smoking have been identified as risk factors. Both low vitamin D levels and cigarette smoking are the strongest risk factors [5, 6, 9, 10]. Although many studies have investigated the epidemiology of MS and the association effect of different genetic, environmental factors and lifestyle on this disease [2, 6, 11–21], to the best of our knowledge, no study has assessed the associations effects of covariates, space and time on incidence rate of MS in Fars province through Bayesian spatio-temporal model.

This study models the incidence rate of MS in Fars province in southern Iran over a 26-year- period from 1991 to 2016 for identifying geographic patterns. We simultaneously investigate the association of some covariates such as vitamin D supplementation intake, smoking, body-mass index (BMI) and alcohol consumption on the number of news cases of MS in 29 counties of Fars province, using Bayesian spatio-temporal model.

## Methods

### Study area

**Fig. 1:**
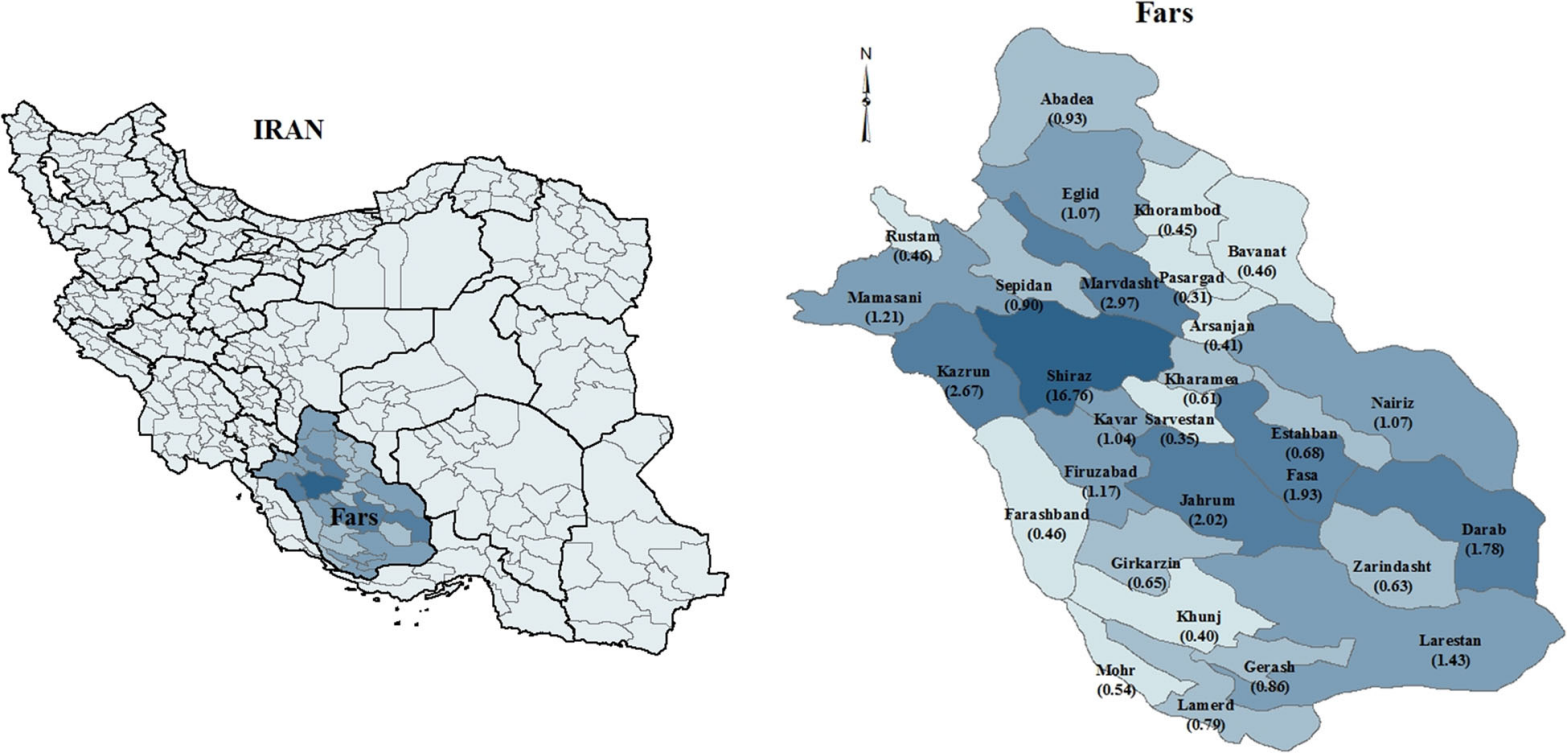
geographical location of Fars province in Iran country (left) and counties located in Fars province (right) (created using ArcGIS Desktop: Release 10.1, https://www.esri.com/en-us/arcgis/about-arcgis/overview)

Fars province is located in the southwest region of Iran (Fig. 1 left) and covers 120,608 km^2^ of land. The province is subdivided into 29 counties and has 4.80 million inhabitants (51% males) according to 2016 census report by the Statistical Center of Iran (SCI) [22]. According to census, Fars population in 1996 was 3,817,036 (1,927,415 males and 1,889,621 females), in 2006 was 4,336,878 (2,204,852 males and 2,132,026 females), and in 2016 it reached 4,851,274 (2,461,251 males and 2,390,023 females). The population at risk in each year was obtained from SCI. To average out, the population at risk (i.e. scaled 1/100,000) in 2006 for the 29 counties is shown in Fig. 1 (right). As shown in the Fig. 1 (right), Shiraz has the highest population at risk.

### Data

This is a historical and retrospective cohort study which was carried out in MS Society of Fars province from 1991 until 2016. This center is the only MS registry center in Fars province, so the information of almost all MS patients is available in its database. All MS patients who fulfilled the McDonald’s criteria [23] from 1991 to 2016 were included in this study. Suspicious cases were referred to the MS committee of Shiraz University of Medical Sciences (SUMS) and were again reevaluated by three expert neurologists to confirm their diagnosis. For all cases, the time at first symptom is defined as time of MS onset. We excluded all patients from other neighboring provinces that were registered in this longitudinal database. In each county, risk factors included: percentage of people that took vitamin D supplements such as calcium vitamin D, cod liver oil and multivitamin not on a regular basis, percentage of smoking population (smoker started smoking before MS onset), percentage of people with abnormal BMI and alcohol consumption (alcohol consumption before MS onset) in the population within the period of 1991–2016. Information about risk factors, based on sampling method proportionate to the population in each county, were obtained from the STEPS reports of Non-communicable diseases (National Institute of Health Research of Iran, https://nihr.tums.ac.ir/) and report annual risk factors in each county in Fars province which are available in Shiraz University of Medical Sciences database.

### Statistical analysis

In this study, the geographical variations of MS incidence rate in 29 counties were analyzed. Standardized Incidence Rate (SIR) was calculated for each county, using the direct method. The observed number of new MS cases in a geographic unit (county) was assumed to follow a Poisson distribution. Furthermore, we considered vitamin D intake, smoking status, BMI, and alcohol consumption as contributing factors. For the assessment of spatial effects and the risk factors on relative risks data were analyzed. Bayesian spatio-temporal model, presented by Bernardinelli et al. [24], was used to identify the temporal pattern of MS incidence rate. Bernardinelli et al. [24] extended the BYM model formula, suggested by Besag, York and Mollie [BYM] [25], with a temporal [24, 25] . The spatio-temporal trend model can be written as

$$ {\eta}_{it}=\alpha +{x}_{it}^T\gamma +{v}_i+{u}_i+\left(\beta +{\delta}_i\right)\ast t $$

The BYM and spatio-temporal models are the most popular full Bayesian models that were explained in many references in detail (see Lawson, A [26]. and Blangiardo M, Cameletti M.) [27]. In these models, the intercept (***α***) and fixed effects (rick factors) ( *γ*) were assumed to follow an improper uniform and normal distribution with zero mean and a small variance as prior, respectively. The unstructured effects (***v***_***i***_), the structured spatial (*u*_***i***_) effects and interaction between space and time (*δ*_*i*_) are random effects and the precision parameters, controlling the amount of variability for the random effects, were assumed to follow a gamma distribution (0.50, 0.0005), as suggested by Bernardinelli et al. [24].

Where incidence, prevalence and confidence intervals (Confidence Interval for a Poisson distribution) were calculated using STATA Software [28]. The spatio-temporal model was coded in the OpenBUGS version 3.2.3 [29] for estimating parameters (see codes of spatial and spatio-temporal models-page 129- in Lawson et.al [30] and ArcGIS 10.1 [31] was used to display results on maps. We ran two chains with 1000 samples as burn-in and 10,000 samples as iteration. Convergence for the chains were confirmed by auto-correlations, trace and densities plots [32].

## Results

We identified 5468 new MS cases (4344 (79%) women and 1124 (21%) men) in Fars province from 1991 to 2016. A total of 3664 patients (67%) were from the city of Shiraz, the capital of Fars province.

Table 1 shows the number of new MS cases in each year, incidence rate per 100,000 populations, age, and the number of females and males in each year in Fars province during 26 years. The highest and lowest incidence rates were observed in 2014 as 11.31(95% Confidence Interval (CI) (10.39, 12.30) per 100,000) and 1992 as 0.22 (95% CI (0.11, 0.44) per 100,000). In addition, the highest and lowest age means were observed in 2014 (32.11 ± 9.40) and 1993 (25.12 ± 8.73). The highest and lowest female/male ratios were observed in 2006 (5.86) and 1991(1.44).

**Table 1:** Incidence of MS per 100,000 persons in the county levels of Fars province in the south of Iran from 1991 to 2016

Year	No. of cases	Incidence rate (95% CI)	Age at onset (y)		No. of cases Female/male	F/M ratio
			Mean ± SD	Min-max		
1991	22	0.61 (0.40,0.93)	28.45 ± 8.11	(10–45)	13/9	1.44
1992	8	0.22 (0.11,0.44)	30.30 ± 8.08	(22–44)	6/2	3.00
1993	32	0.86 (0.61,1.22)	25.12 ± 8.73	(8–44)	21/11	1.91
1994	28	0.75 (0.52,1.08)	26.89 ± 8.25	(11–43)	18/10	1.80
1995	35	0.92 (0.66,1.28)	27.32 ± 8.09	(16–46)	29/6	4.83
1996	35	0.91 (0.65,1.27)	27.43 ± 8.28	(12–46)	31/4	7.75
1997	26	0.67 (0.45,0.98)	25.24 ± 5.89	(14–40)	21/5	4.20
1998	48	1.22 (0.92,1.61)	31.51 ± 12.8	(13–77)	39/9	4.33
1999	77	1.93 (1.54,2.41)	28.21 ± 8.43	(14–48)	59/18	3.28
2000	77	1.90 (1.52,2.38)	29.44 ± 8.77	(9–48)	62/15	4.13
2001	91	2.22 (1.81,2.73)	28.01 ± 8.50	(8–55)	77/14	5.50
2002	104	2.51 (2.07,3.04)	28.20 ± 8.42	(14–53)	86/18	4.78
2003	119	2.83 (2.37,3.39)	29.09 ± 9.03	(11–56)	106/13	8.15
2004	133	3.13 (2.64,3.71)	28.58 ± 8.46	(10–59)	101/32	3.16
2005	199	4.63 (4.03,5.32)	28.57 ± 8.06	(13–53)	169/30	5.63
2006	199	4.57 (3.98,5.26)	28.82 ± 8.62	(12–59)	170/29	5.86
2007	228	5.18 (4.55,5.90)	31.23 ± 10.80	(7–86)	186/42	4.43
2008	279	6.27 (5.58,7.05)	30.09 ± 8.98	(9–55)	221/58	3.81
2009	288	6.40 (5.70,7.18)	30.47 ± 9.01	(10–56)	237/51	4.65
2010	391	8.59 (7.78,9.49)	29.77 ± 8.64	(8–63)	323/68	4.75
2011	438	9.52 (8.67,10.46)	31.49 ± 9.45	(5–69)	366/72	5.08
2012	488	10.49 (9.60,11.47)	30.26 ± 8.84	(7–64)	405/83	4.88
2013	510	10.85 (9.95,11.83)	30.80 ± 9.08	(9–60)	397/113	3.51
2014	537	11.31 (10.39,12.30)	32.11 ± 9.40	(11–64)	432/105	4.11
2015	533	11.10 (10.20,12.09)	32.09 ± 9.27	(8–62)	415/118	3.52
2016	543	11.20 (10.29,12.18)	32.43 ± 8.82	(11–62)	354/189	1.87

Age-specific prevalence rates of MS are shown in Table 2. The highest and lowest prevalence rates belonged to 20–30 (362.10 (95% CI, (347.31, 377.28) per 100,000) and > 60 (4.60, 95% CI, (2.61, 7.59) per 100,000) age group. A statistically significant difference was determined between the prevalence of MS among men and women (*P* < 0.0001), except age group> 60. Highest and lowest values of MS Female/male ratio were observed in age groups < 20 (4.65) and > 60 (1.50).

**Table 2:** Age-specific prevalence rates of MS in the county (per 100,000 persons) of Fars province in the south of Iran from 1991 to 2016

Age group	No. of cases	Prevalence	No. of cases Female/male	F/M ratio	*P*-value F to M
< 20	689	97.61 (90.40–105.19)	567/122	4.65	<0.0001
20–30	2261	362.10 (347.31–377.28)	1797/464	3.87	<0.0001
30–40	1629	233.02 (221.77–244.60)	1301/328	3.97	<0.0001
40–50	742	167.10 (155.89–179.60)	565/177	3.19	<0.0001
50–60	132	40.01 (33.40–47.32)	105/27	3.89	<0.0001
> 60	15	4.60 (2.61–7.59)	9/6	1.50	0.6056

**Fig. 2:**
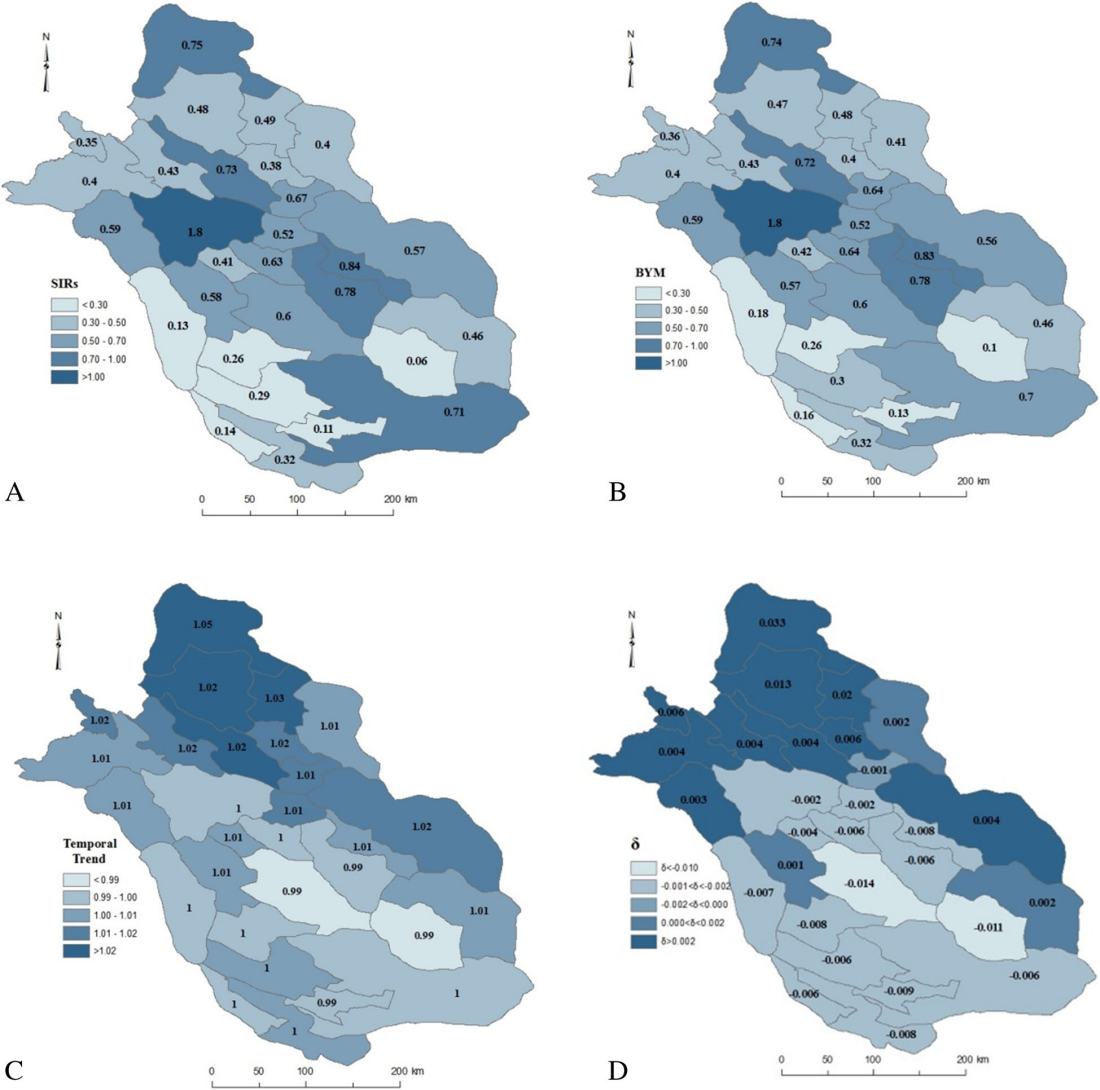
MS incidence rate across the 29 counties of Fars province with classic SIR (**A**), posterior mean of the spatial main effect (relative risk) by BYM model (**B**) posterior temporal trend of MS incidence rate (**C**), and posterior mean of the differential time effect *δ*_*i*_ (**D**), (created using ArcGIS Desktop: Release 10.1, https://www.esri.com/en-us/arcgis/about-arcgis/overview)

We generated maps of geographical variations of MS incidence across the 29 counties of Fars province with classic SIR (Fig. 2A), BYM model (Fig. 2B) spatiotemporal trend model (Fig. 2C), and posterior estimate value of *δ*_*i*_ (Fig. 2D). *δ*_*i*_ shows the difference between the global trend of incidence rate and the area-specific trend of incidence rate (*δ*_*i*_ < 0, shows that this trend is less steep than the mean trend, while *δ*_*i*_ > 0 shows that the area-specific trend of incidence rate is steeper than the mean trend). The maps should be interpreted by considering that different shades are proportional to the incidence rate value. In other words, darker areas have the higher incidence rate. The averages of SIRs and relative risks were 0.52 and 0.43, respectively. As can be seen in Fig. 2 (A and B), Shiraz is at higher risk than other counties. Shiraz had the highest SIR and relative risk for MS with values of 1.80 and 1.80 (standard deviation = 0.03), respectively. Zarindasht county in southeast of Fars province had the lowest SIR and relative risk with values of 0.06 and 0.11 (standard deviation = 0.03), respectively. Figure 2C shows the temporal trend of the incidence rate across different regions of Fars province during the period of 1991–2016. The estimated value of time coefficient in this model was 0.0075. The exponential corresponding to the coefficient of time is equal to 1.007 for each successive year. This finding shows that although there is an increasing trend of incidence in Fars, it is very uniform. Figure 2D shows the change of temporal patterns of MS incidence rate. Darker areas show that the trend is steeper for those counties compared with the global trend and brighter areas show that the trend is less steep than the global trend.

Another important point that can be inferred from Fig. 2C is the steady increase of incidence rate of relative risk over time in the northern regions of Fars province. Figure 2B depicts the average over a period of 26 years, showing higher overall incidence rates in the central regions of Fars. On the other hand, Fig. 2C incorporates relative changes over this time period, showing a geographical shift of the incidence rate from the central regions to the northern regions of the province.

Table 3 is a summary of the statistics related to different covariates used in BYM model. The estimated fixed effects show that the percentages of vitamin D intake is negatively associated with MS incidence while the percentage of people with smoking status is positively associated with MS incidence. It was found that 1% increase in vitamin D supplementation intake is associated with 2% decrease in the risk of MS incidence whereas 1% increase in smoking is associated with 16% increase in the risk of MS incidence.

**Table 3:** Summary statistics: posterior mean, posterior standard deviation (SD) and posterior 95% credibility interval for the fixed effects of BYM models in MS incidence

Covariate	Coefficient (SD)	Exp (coefficient)	95% credible Interval
**% of vitamin D supplementation intake**	−0.02 (0.01)	0.98	(−.02,-0.01)^*^
**% of smoking**	0.15 (0.06)	1.16	(0.03,0.24)^*^
**% of normal BMI**	0.02 (0.02)	1.02	(−0.02,0.02)
**% of Alcohol consumption**	1.01 (0.78)	2.75	(−0.51,2.58)

## Discussion and conclusion

This study spatially evaluated the MS incidence distribution in Fars province, using Bayesian model. Spatial description of the disease is a useful tool for evaluating the incidence rate data and could lead to further public health investigations and interventions [33]. As far as we know, this is the first study that has estimated the spatio-temporal incidence rate of MS in Fars province. This study also investigates the association of vitamin D supplementation intake, smoking, BMI, and alcohol consumption with MS incidence rate as well as spatial dependence between the neighboring regions. The findings suggest that further studies are warranted in order to confirm the allocations of health resource across different region.

In the current study, the average of Female/male ratio was 4.25 and women had higher risk than men. Among the age groups, the highest prevalence belonged to range 20–30 (362.10 per 100,000-person), and then 30–40 years old (233.00 per 100,000-person). Our results are similar to Sahraian et al.’s study [34] on 8000 cases of MS in 2010. They reported that the most cases of MS were in the age range of 20–39 years old and Iranian women have higher risk of MS. The results of spatio-temporal model describe the low incidence rate of MS in south and southeast of Fars province. Zarindasht county, located in south with a rather hot and dry climate and high temperature, had the lowest incidence rate. This result is in line with previous studies that showed maintaining adequate levels of vitamin D (as one of the environmental factors) has a protective effect. It also results in lower risk of developing autoimmune diseases including MS [35–37]. The results of BYM model (without time effect) in this study showed that larger counties, located in the center of Fars province, especially Shiraz and Abadeh in northeast, had high MS incidence rates. This is in concordance with previous studies which showed MS to be more common among urban dwellers than rural ones [38]. There is no clear explanation as to why, but Dehghani et al. [39] introduced urbanization as a major risk factor in incidence rate of MS in Iran. In addition, Sahraian et al. [40] indicated that the impact of air pollution, unhealthy lifestyle, exposure to industrial solvents, low exposure to sunlight, infectious agents and smoking have increased incidence rate of MS among urban dwellers.

Our results showed that the percentage of vitamin D supplementation intake and smoking are associated with the risk of MS incidence which is in line with previous studies that had suggested that maintaining adequate levels of vitamin D could have a protective effect by lowering the risk of developing autoimmune diseases including MS [35–37]. When a person has MS, his or her immune system attacks the coating that protects nerve cells. Research suggests a significant positive association of vitamin D on the immune system [35–37, 41]. Smoking as a risk factor can influence the course of MS [16, 42, 43]. Both the duration and intensity of smoking can contribute independently to the increased risk of MS [44]. Consistent studies on MS patients and healthy controls have provided evidence that both active and passive smoking can result in an increased risk of MS and disease progression. Studies have shown that the risk of MS associated with HLA genotypes is increased by smoking status [42, 45].

According to several studies, obesity is one of the potential risk factors of MS. The growing world-wide obesity epidemic has multiple deleterious effects on public health and has also been associated with an increased risk of MS [46]. Increased obesity leads to lower levels of 25-hydroxyvitamin D which in turn predisposes to MS [47]. The association between obesity and MS is similar among men and women, and the observed trend of higher BMI resulted in a higher risk of developing MS [48]. However, in our study BMI had no association with the incidence of MS.

Although the impact of alcohol, which might directly suppress various immune responses, on the risk of developing MS has been investigated in different studies, the results were inconsistent. In some studies, researchers found no association between alcohol intake and the risk of MS [49]. But some studies showed a potential dependency between alcohol consumption and the incidence of MS [10].

## Conclusion

Bayesian spatio-temporal analysis of MS incidence rate revealed that the trend in the south and south east of Fars province is less steep than the mean trend of this disease. The lower incidence rate was associated with a higher percentage of vitamin D supplementation intake and a lower percentage of smoking. Previous studies have also shown that smoking and low vitamin D, among all covariates or risk factors, might be associated with high incidence of MS.

Our study was a long-term study in an almost uniform population but it had some limitations which should be taken into consideration in future studies. The major limitation in such studies is the incomplete registration of information due to lack of diagnosis facilities and access to neurologist, especially in rural areas. However, the various models in disease mapping, such as Bayesian hierarchical models, for smoothing disease rates and solving this problem have been suggested. Bayesian hierarchical models try to improve the estimates of log risk by using neighbor regions information in a spatial structured component as well as region variation in an unstructured component. Despite these models, government supports for the complete recording of information could increase the accuracy of the estimates.

Another limitation is related to risk factors. We needed covariates in each area during study for spatio-temporal modeling. However, some covariates such as climate, sunlight, air pollution industrial solvents and so on, were not available per year between 1991 and 2016; hence, we had the information of four covariates during this period. We are hoping, by improving the quality of data registration in Iran in recent years, to investigate environmental causes by statistical analysis very soon.

## Data Availability

The datasets are available from the corresponding author upon reasonable request.

## Abbreviations

MS: Multiple Sclerosis
RR: Relapsing- Remitting
SP: Secondary Progressive
PP: Primary Progressive
PR: Progressive Relapsing
BMI: Body-Mass Index
SCI: Statistical Center of Iran
SUMS: Shiraz University of Medical Sciences
STEPS: .
SIR: Standardized Incidence Rate
BYM: Besag, York and Mollie
CI: Confidence Interval
SD: Standard Deviation
